# Social Study Resources and Social Wellbeing Before and During the Intelligent COVID-19 Lockdown in The Netherlands

**DOI:** 10.1007/s11205-021-02654-2

**Published:** 2021-03-10

**Authors:** Llewellyn Ellardus van Zyl

**Affiliations:** 1 Department of Industrial Engineering, University of Eindhoven, Eindhoven, The Netherlands; 2 Optentia Research Focus Area, North-West University (VTC), Vanderbijlpark, South Africa; 3 Department of Human Resource Management, University of Twente, Enschede, The Netherlands; 4 Department of Social Psychology, Institute for Psychology, Goethe University, Frankfurt am Main, Germany

**Keywords:** Social wellbeing, Social study resources, University students, COVID-19, Piecewise latent growth modelling, Coronavirus

## Abstract

The first intelligent COVID-19 lockdown resulted in radical changes within the tertiary educational system within the Netherlands. These changes posed new challenges for university students and many social welfare agencies have warned that it could have adverse effects on the social wellbeing (SWB) of university students. Students may lack the necessary social study-related resources (peer- and lecturer support) (SSR) necessary to aid them in coping with the new demands that the lockdown may bring. As such, the present study aimed to investigate the trajectory patterns, rate of change and longitudinal associations between SSR and SWB of 175 Dutch students before and during the COVID-19 lockdown. A piecewise latent growth modelling approach was employed to sample students’ experiences over three months. Participants to complete a battery of psychometric assessments for five weeks before the COVID-19 lockdown was implemented, followed by two directly after and a month follow-up. The results were paradoxical and contradicting to initial expectations. Where SSR showed a linear rate of decline before- and significant growth trajectory during the lockdown, SWB remained moderate and stable. Further, initial levels and growth trajectories between SSR and SWB were only associated before the lockdown.

## Introduction

The diverse biological, genetic, and epidemiological attributes of the Severe Acute Respiratory Syndrome Coronavirus 2 (SARS-CoV-2 or ‘COVID-19’) has made it one of the most contagious diseases in modern history (Wilder-Smith et al. 2020). Considered more lethal and infections than the SARS-CoV or MERS-CoV viruses (Meo et al. 2020), COVID-19 rapidly spread across the globe, infecting more than 51 million people, and resulted in more than 2 million deaths since its first diagnosis on the 12th of December 2019 (Sahin et al. 2020). With the absence of validated treatment strategies or vaccines, the only effective public health intervention to manage transmittable diseases is to control person-to-person infections through social distancing, isolation, quarantine, and community containment procedures (Cetron and Simone 2004; Masters et al. 2020; Wilder-Smith and Freedman 2020; Wilder-Smith et al. 2020). These non-pharmaceutical interventions’ (NPIs) focus on a suite measures to both mitigate possibilities of infection and suppress the spread of the disease to prevent deaths, health care system overloads, and to reduce incidence (Chowdhury et al. 2020).

Since declared a global pandemic on the 11th of March 2020, many countries started to adopt and implement different forms of NPIs to flatten the proverbial COVID-19 ‘infection curve’ (Chowdhury et al. 2020; RIVM 2020b). Within Italy, Spain and France, governments opted to implement strict national lockdown procedures where citizens were forced to stay at home, and all non-essential travel and exposure to the external environment were prohibited (de Haas et al. 2020). In other countries such as Sweden and the Czech Republic, less restrictive (or ‘soft lockdown’) measures were implemented allowing citizens to still visit restaurants/bars, children to go to school and allowing people to live their lives in a fairly non-disruptive manner (Kavaliunas et al. 2020). Within the Netherlands, the Dutch Government implemented a middle of the road NPI strategy which it called an “*Intelligent COVID-19 Lockdown*” (de Haas et al. 2020). This approach involved a case-based isolation strategy, coupled with internal travel restrictions, social isolation, social distancing, self-quarantine, and public event cancellations (Fried 2020). Citizens were urged to stay at- and work from home as much as possible, and visits to nursing homes were prohibited (Dutch Government 2020; RIVM 2020a). Further, large-scale business-, school-, and university closures followed, and international travel was restricted (de Haas et al. 2020; Fried, 2020). Despite these restrictions, citizens were still permitted to move around freely and meet with social contacts under the condition that they maintained a 1.5 m distance (Chorus et al. 2020; de Haas et al. 2020).

Although this ‘*Intelligent COVID-19 Lockdown*’ significantly blunted the peak of infections during the first wave and alleviated the pressure off the public healthcare system, the societal impact was severe (de Haas et al. 2020). The measures resulted in large scale unemployment, and a major decline in the Dutch economy (Van Zyl et al. 2021). Further, radical changes in individuals’ activity patterns, the way they worked, studied shopped, and connected to others occurred (Antonides and van Leeuwen, 2020). As the realities of the measures started to set in, it was argued that individuals may start to experience chronic loneliness, and -boredom which in turn leads to depression, general (mental) health issues, irrational decision making and even suicide (Banerjee and Rai 2020). Long term social isolation may even affect brain structures, increase social monitoring, and erode social bonds (Cacioppo and Cacioppo 2020; Gardner et al. 2005). As such, many scholars and social welfare agencies warned that the lockdown measures would have long-lasting adverse effects on the *social wellbeing* (SWB) and mental health of individuals; particularly for vulnerable groups (Fried 2020; de Haas et al. 2020 Pancan et al. 2020).

Although the negative consequences of the lockdown could affect anyone, certain population groups are more vulnerable to onset than others (Keyes 2002). Research suggests that university students are three times more likely to the onset of psychopathology and mood disorders due to social isolation and loneliness than the general public (Auerbach et al. 2016). They are therefore considered a vulnerable group (Fried 2020; Ribeiro et al. 2017) and need specific resources to cope with the psychological consequences of the COVID-19 pandemic (Capone et al. 2020).

Students are thus academically- and psychologically dependent upon the social resources provided at university (Cilliers et al. 2018). Peer- and Lecturer support provide a means through which to buffer against the negative impact that both life and studies have on their mental health and academic performance (Lesener et al. 2020; Mokgele and Rothman 2014). Demand for these “*study-related social resources*” (SSR) increase dramatically during times of uncertainty (Capone et al. 2020; Leigh-Hunt et al. 2017; Mtshweni 2019) as it provides students with a means to cope with the associated stressors and anxiety that radical change brings. If students report positive relationships with their peers and feel as though their lecturers support their professional growth, they are more likely to be engaged, they perform better on formative assessments and are less likely to drop out of university (Cilliers et al. 2018; Keyes, 2005). In contrast, when students feel a lack of support from peers/lecturers, their perceptions of study related demands increase which in turn has a negative effect on both academic performance and overall mental health (Cilliers et al. 2018; Capone et al. 2020; Mtshweni 2019). Students’ wellbeing is therefore directly dependent upon the positive relational experiences they have with their peers and lecturers as it provides a means through which to both process study- and life-related demands as well as manage the radical changes and uncertainty created by COVID-19 (Capone et al. 2020).

A major cause of uncertainty reported by Dutch students during the first COVID-19 lockdown was how it would affect their educational trajectories at university (Fried 2020). The COVID-19 lockdown radically altered the tertiary educational system within the Netherlands which directly affected students (Fried 2020). Not only did the measures remove access to students’ SSR but it also denied access to important physical resources needed to complete their studies (e.g. libraries closed, cancelled lectures, delayed exams and restricted access to virtual private networks) (Maastricht, 2020; TU/e 2020). Despite already having to cope with the radical changes in “normal life”, students were confronted with fundamental shifts in their educational routines. Students needed to adapt to online education, examinations required virtual proctoring, contact with lecturers/peers were severely limited, assignment/examination formats changed, and they were bombarded with conflicting information from various sources (de Haas et al. 2020; Fried 2020; Maastricht 2020; TU/e 2020). These changes, the uncertainty and confusion about educational activities, the developing fear caused by the ever-increasing COVID-19 mortality rates, and the implementation of lockdown procedures, may significantly increase students’ need for the social support, certainty and emotional containment which SSR provide (Fried 2020; Roy et al. 2020). The lack of SSR, coupled with the psychological impact of the COVID-19 lockdown measures and the associated social isolation may result in a significant increase in psychological distress (Brooks et al. 2020). This, in turn, may have compounding negative effects on students’ SWB (Pancani et al. 2020) and mental health (O’Regan 2020).

It is therefore imperative to investigate how SSR and SWB developed before, and during the ‘intelligent COVID-19 Lockdown’ within the Netherlands. Understanding how SSR and SWB developed before and during the lockdown procedures is critically important as it may inform the policies and interventions that universities may employ to protect students’ mental health during the COVID-19 pandemic. As such, the present study aimed to investigate the longitudinal growth trajectories and associations between SSR and SWB within a sample of Dutch master students before and during the COVID-19 lockdown.

## Literature Review

### Social Wellbeing of University Students

The COVID-19 pandemic has a significant effect on the social wellbeing of people, as it limits the extent towards which individuals’ social needs can be met (Fried 2020). Social wellbeing is an important dimension of both physical and mental health in times of crisis (Pancani et al. 2020) and plays a vital role in ensuring the optimal functioning of society (Zhang and Ma 2020). Keyes (2002) argued that SWB is a public phenomenon which emphasizes the fit between the social needs of the individual and their social realities. Drawing from the social psychological and sociological frameworks of Durkheim (1951) and Ryff (1989), Keyes (1998, p 204) argued that social wellbeing refers to the extent to which “*individuals feel they make valued social contributions, view society as meaningful and intelligible, experience a sense of social belonging, maintain positive attitudes toward others, and believe in the potential for society to evolve positively*”. In effect, SWB refers to individuals’ subjective judgment of the quality of their social relationships, the efficacy of their interaction with social institutions/communities and how others respond to them (Keyes et al. 2020).

From this perspective, Keyes (2002) argued that SWB is a function of five inter-related factors: (1) social integration (the extent towards which one is integrated into the community), (2) social contribution (that one is a vital part and make a valuable contribution to society), (3) social coherence (the believe that society functions as an integrated and coherent whole, of which one is an active member), (4) social actualisation (viewing society as having potential to grow and develop through its citizens), and (5) social acceptance (showing trust towards others and viewing people as naturally good). These factors describe the extent towards which individuals can overcome social challenges and function effectively in their social communities (Keyes et al. 2020). Self-determination theory proposes that SWB is influenced by the environmental conditions in which individuals are nested and can only occur when one's basic psychological needs (autonomy, competence and relatedness) are met (Ryan 2009; Ryan and Deci 2000). SWB is therefore not a stable trait, but an internal capacity that develops or changes in relation to societal/cultural values and environmental demands (Goodenough and Waite 2020).

SWB is also associated with various positive physical and mental health outcomes in student populations. When students are deeply rooted in their study communities and feel a shared sense of belonging to or have strong social bonds with peers, they are more effective in managing environmental demands, they perform better on formative assessments and are generally happier and healthier (Howell 2009; Seligman 2012). However, research has shown that more than half the world’s population suffers from poor SWB (Rashid and Seligman 2018; Seligman 2012) and that students do not have the necessary social resources in place to effectively manage such (Oritz-Ospina and Roser 2020). This, in turn, may have a significant effect on their long term mental health.

Due to poor levels of SWB, university students are at a higher risk of developing lasting psychopathological disorders (Blanco et al. 2008; Seligman 2012). Research suggests that one in three students report severe levels of psychological distress stemming from social isolation, loneliness, poor social contact and lower levels of SWB (Blanco et al. 2008; Eisenberg et al. 2013; Seligman 2012). This is as a result of ever-increasing study-related stressors such as an intensive educational programme, strict deadlines (time pressure), poor relationships with fellow students and lecturers, (Basson and Rothmann 2019; Houghton et al. 2012), high levels of social comparison, peer pressure, study-life imbalances (Bergin & Pakenham, 2015) and drastic life changes like living away from their families (Blanco et al. 2008). Further, during pandemics, the SWB of students could have adverse effects on physical health, life achievement, personal relationships, and life satisfaction as social isolation, fear of infection and uncertainty takes its toll (Brooks et al. 2020; Lau et al. 2005). These problems lead to significant impairment in psychological functioning which in turn negatively affects social cognition, academic performance, -throughput and learning potential (Ebert et al. 2018). Researchers have argued that students are unable to effectively manage these demands, and do not have the required personal, or SSR at university to buffer against the effects thereof on their SWB and academic performance (Mokgele and Rothmann 2014). Although the antecedents and outcomes of SWB are clear within the literature, the specific factors and the extent towards which these would be applicable during the COVID-19 pandemic is not known (Fried, 2020; Mertens et al. 2020).

### Social Study Related Resources of University Students

The Study Demands-Resources Framework (SDRF; Lesener et al. 2020; Mokgele and Rothmann 2014) provides an interesting lens through which to interpret the factors that influence students SWB during the COVID-19 Pandemic. The SDRF is rooted in the well-known Job Demands-Resources Model (c.f. Demerouti et al. 2000) and claims that specific work/study-related characteristics lead to wellbeing and academic performance. Study characteristics refer to the context-specific study demands and study resources available to students. From this perspective, *Study Demands* are defined as the factors that require students to expert high levels of cognitive/physical/emotional effort over a sustained period (Mokgele & Rothmann, 2014). *Study resources* on the other hand refer to the factors that promote study engagement and guards against the development of common mental health problems (Cilliers et al. 2018). Study resources can also be classified into functional study-related resources (e.g. information availability and growth opportunities) and social study resources (e.g. peer support and lecturer support) (Van Zyl and Rothmann 2012). Lesener et al. (2020) argued that when study demands are high, that it activates a health impairment process through factors such as burnout. This in turn affects overall (social) wellbeing and results in poor academic performance. In contrast, an abundance of study related resources activates a motivational process that enhances engagement and improves overall (social) wellbeing (Lesener et al. 2020).

Mokgele and Rothmann (2014) argued that a lack of SSR has a direct impact on the SWB and mental health of students. When students lack SSR they cannot reduce the potentially harmful influence of study demands on their social wellbeing, which will lead to an inability to adequately perform (Robins et al. 2015). In contrast, resource availability enhances study engagement which in turn strengthens SWB, enhances learning potential (Cilliers et al. 2018) and leads to overall physical health. Several studies have shown that specific SSR (i.e. peer support, lecturer support) may enhance the SWB of students (Mokgele & Rothmann, 2014). Students' social inclusion into the university environment is fostered through lectures, discussions with peers, campus involvement, and learning communities (Basson and Rothmann 2019). Therefore, social support mechanisms like these are important resources needed to aid students in managing both their daily lives but also to cope with the challenges which the COVID-19 lockdown brings.

It is important to note that given the radical changes in the educational system during the COVID-19 lockdown, students’ perceptions of available SSR may be different than from normal circumstances (Fried 2020). Given that students are not able to physically meet with their peers, or access limited to lecturers, they may report lower levels of available SSR. Further, the established association between SSR and SWB may also be affected due to the sudden and radical changes in both the educational system and general life. It is therefore not clear how SSR may affect SWB during the COVID-19 lockdown. As such, it’s important to investigate how SSR and SWB developed and are related before and during the COVID-19 lockdown. Understanding the growth trajectories of SSR and SWB as well as their association will aid universities in designing effective interventions to manage the effect the COVID-19 pandemic may have on students’ mental health.

### Current Study

The purpose of this paper was to investigate the growth trajectories and rates of change of SSR and SWB of Dutch master students before and during the COVID-19 Lockdown. Further, the aim was to determine if and how the association between SR and SWB changes before and during the COVID-19 lockdown. Although a clear hypothesis can be formed as to the positive relationship between SSR and SWB under normal circumstances, no literature is available that specifically explains such *during* pandemics. As such, no a priori hypotheses can be developed. However, it was expected that both SSR and SWB are negatively affected by the COVID-19 lockdown procedures.

## Research Method

### Research Approach and Procedure

The longitudinal trajectories and association between SSR and SWB of master students at a Dutch University were investigated through employing a longitudinal electronic survey-based research design. Participants in this study were first-year master students registered for a course on Research Methodology between January and April 2020.

Before the start of the course, students were invited to take part in the study via an introductory email. This email described the purpose of the study and the research procedure. Further, it highlighted the voluntary nature of participation, discussed their rights and responsibilities, it guaranteed confidentiality and anonymity, and mentioned that they could withdraw from participation at any time. A separate email box was created and managed by an external research partner, whereby participants could direct any questions or queries they may have had about the study or to discuss any challenges/problems they experienced throughout. After agreeing to participate, participants were sent a separate email with further instructions on how to complete the questionnaires. All guidelines for Ethical Research Practices by the American Psychological Association as well as local legislation were strictly followed.

Data was collected over a period of three months and required participants to complete seven weekly electronic self-assessments and a final assessment a month later. All questionnaires were distributed electronically, and participant responses were linked via a unique code. The first four weekly measures took place before the COVID-19 lockdown procedures were implemented. The fifth assessment occurred in the week where the lockdown procedures were announced and implemented. The sixth and seventh weekly assessment took place directly after the lockdown procedures were implemented. The eight (final) assessment occurred one month after the seventh assessment. The data was collated, captured, and stored on a secured server in compliance with the research institution’s data management policy.

Data quality was also managed through the implementation of several *Attention Checks*. Two of Abbey and Meloy’s (2017) guidelines for attention checks were implemented. Firstly, direct queries were inserted into the instructions of two sections of the questionnaire (e.g. “Please rate item 7 on the scale as Completely Disagree” and “Write the word sky in the textbox and rate it as Absolutely”). Secondly, a post hoc analysis of the response patterns, response consistency and time taken to complete the questionnaire was implemented (Buchanan and Scofield 2018). If a participant did not accurately complete both attention checks, their response to the given assessment was removed.

## Participants and Power

To determine the most appropriate sample size to elicit the desired effect for the LGM estimations, a power analyses using the Satorra-Saris method was used (c.f. Wong and Wong 2020, p. 446 for a non-technical primer). This method was estimated in Mplus v. 8.4, where the intercept of the population mean for SSR and SWB was specified to be 0.2 and a variance of 0.3. A linear growth trajectory was assumed where time was coded to correspond to the weeks in which assessments took place: 0, 1, 2, 3, 4, 5, 6, 10. Further, the rate of change (i.e. the latent slope growth factor) was assumed to be 0.1 with a variance of 0.1. An initial, potential sample size was set at 50. This model was run and it produced an estimated noncentrality parameter of *λ* = 4.552. This was used to compute the statistical power required to detect the effect at an α level of 0.05. The same model was then run multiple times, increasing the potential sample size by 10, up until 175. These estimated noncentrality parameters and their corresponding potential sample sizes were then used as inputs to estimate overall statistical power (c.f. Table 6 for a full overview). The results showed that a sample size of *N* = 120 was needed to have a power greater than 0.80 to detect a rate of change of 0.10 in both latent growth models.

As such, a population-based census sample of 175 master students registered at a Dutch University was drawn to compensate for the inevitable sample attrition/dropout. Data was collected over a three-month period (January to April 2020) and required participants to complete a battery of psychometric assessments for five weeks *before* the COVID-19 lockdown was implemented, followed by two directly *after*. The final assessment took place a month after the previous assessment. Lockdown procedures were introduced in week 5.

Most of the participants were Dutch-speaking (94.3%), Dutch national (94.3%) males (66.3%) between the ages of 22 and 25 years old (94.3%) enrolled for a master’s program at a University in the Netherlands (*c.f.* Table 1). All participants were residing in the Netherlands during the study.

**Table 1:** Characteristics of participants (*n* = 175)

Item	Category	Frequency (*f)*	Percentage (%)
Gender	Male	116	66.3
	Female	58	33.1
	Other	1	0.6
Age (years)	22–25 years	165	94.3
	26–30 years	10	5.7
Nationality	Dutch	165	94.3
	Other	10	5.7
Home language	Dutch	165	94.3
	Other	10	5.7

### Measures

The *Study Resources Scale* (Mokgele and Rothmann 2014) was used to measure the availability of social study resources. Two subscales of the instrument were used to measure: *peer-support* (3 items: e.g. ‘When necessary, can you ask fellow students for help?’) and *lecturer-support* (8 items: e.g. ‘Can you discuss study problems with your lecturers?’). Participants were requested to reflect upon the preceding week and rate items on a 5-point Likert scale ranging from 1 (“Never”) to 5 (“Always”). The scale showed to be a reliable instrument across all 8-time points in this study with Cronbach’s ranging from 0.84 to 0.96.

The *Social Wellbeing Subscale* of the *Mental Health Continuum Short-Form* (Keyes 2005) was used to measure overall SWB. It consists of five self-report items, rated on a 6-point Likert scale ranging from 1 (“Never”) to 6 (“Every Day”). Participants were requested to reflect upon the preceding week and indicate to what extent they experienced social wellbeing (e.g. ‘*that the way in which our society functions, makes sense to you’*). The scale showed to be a reliable instrument across all eight-time points in this study with Cronbach’s ranging from 0.76 to 0.85.

### Data Analysis

Both SPSS v.26 (IBM, 2020) and Mplus v.8.4 (Muthén and Muthén 1998–2020) were used to process the data. *First*, the distribution of the data, the level of internal consistency and the relationships amongst the factors were assessed through descriptive statistics (means, standard deviations, skewness and kurtosis) and Pearson/Spearman correlations. Skewness and Kurtosis ranging between − 2 and + 2 were used as indicators of multivariate normality (FIeld, 2020). Further, internal consistency for the instruments were established through both Cronbach’s alpha (lower-bound: α > 0.70; Nunnally and Bernstein 1994) as well as the point-estimate composite reliability (upper-bound: *ρ* > 0.80; Wong & Wong, 2020). Practical significance for Pearson/Spearman’s correlation coefficients were established when relationships were statistically significant (*p* < 0.05) and effect sizes were either small (*r* > 0.10), medium (*r* > 0.30) or large (*r* > 0.50) (Steyn 1999, 2002).

*Second*, through structural equation modelling (SEM) with the robust maximum likelihood (MLR) estimator, a series of unconditional Latent Growth Models (LGM) were estimated to determine the intercept and slopes of students’ SSR and SWB. A sequential and competing measurement model process was employed to determine the best fitting LGM. First, an intercept only model for each factor was estimated. Thereafter separate LGM for linear-, quadratic-, and piecewise growth trajectories were calculated to determine the best-fitting model for the data. For the piecewise LGM, two separate linear growth factors were estimated representing the slopes *before* (Week 0–4) and during (Week 5–10) the COVID-19 lockdown. Time 4 represented the interior **knot** (Wang and Wang 2020). The first and second growth trajectories were constrained to [0,1,2,3,**4**,4,4,4] and [0,0,0,0,**0**,1,2,6]. Piecewise LGM is used when one wants to compare the growth trajectories of a factor between two substantial periods of interest (Duncan et al. 2013). Model fit was determined through conventional SEM standards and fit indices used to compare competing LGMs (*c.f.* Table 2, adapted from Wong and Wong 2020).

**Table 2:** Model Fit Statistics. Adapted from Wong and Wong (2020)

Fit indices	Cut-Off Criterion	Sensitive to N	Penalty for model complexity
*Absolute fit indices*			
Chi-Square (χ^2^)	Lowest comparative value between measurement or latent growth models Significant (p > 0.01)	Yes	Yes
*Approximate fit indices*			
Root-Means-Square Error of Approximation (RMSEA)	< 0.08 but > 0.01 90% CI Range doesn’t include Zero	Yes	Yes
Standardized Root Mean Square Residual (SRMR)	< 0.08 but > 0.01	Yes	No
*Incremental fit indices*			
Comparative Fit Index (CFI)	> 0.90 but < 1.00	No	Yes
Tucker-Lewis Index (TLI)	> 0.90 but < 1.00	No	Yes
Akaike information criterion (AIC)	The lowest value in comparative measurement or latent growth models	No	No
Bayes information criterion (BIC)	The lowest value in comparative measurement or latent growth models	No	No

*Finally*, a sequential piecewise multi-process LGM was employed to simultaneously model the growth processes and longitudinal associations between SSR and SWB. Here, the intercept and slopes of SSR were regressed on those of SWB. Statistical significance was set at *p* < 0.05.

## Results

### Descriptive Statistics, Internal Consistency, and Correlations

Table 3 summarises the descriptive statistics, internal consistencies, and correlation coefficients of all the factors. The table showed that: SSR was not normally distributed (Skewness/Kurtosis > 2; Field 2020), all instruments showed acceptable levels on internal consistency at both the lower- (*α* > 0.70; Nunnally and Bernstein 1994) and upper- bound limits (upper-bound: *ρ* > 0.70; Wong and Wong 2020). Positive relationships were found between all factors (*p* < 0.05) with effect sizes ranging from small (*r* = 0.14) to large (*r* = 0.83).

**Table 3:** Descriptive statistics, Cronbach Alphas and Pearson Correlations (*n* = 175)

Factor	µ	σ	SK	Rku	α	*ρ*	1	2	3	4	5	6	7	8	9	10	11	12	13	14	15	
*Social study resources*																						
1	Week 0	3.61	0.52	− 0.78	2.94	0.74	0.79	–														
2	Week 1	3.55	0.50	− 0.59	0.50	0.79	0.83	0.78	–													
3	Week 2	3.54	0.54	− 0.93	2.71	0.83	0.84	0.79	0.82	–												
4	Week 3	3.56	0.47	− 0.78	3.49	0.77	0.82	0.74	0.77	0.84	–											
5	Week 4	3.56	0.54	− 0.97	3.91	0.82	0.84	0.60	0.66	0.66	0.75	–										
6	Week 5	3.56	0.54	− 0.86	3.04	0.84	0.86	0.64	0.66	0.70	0.80	0.66	–									
7	Week 6	3.57	0.51	− 0.74	3.18	0.84	0.86	0.62	0.70	0.71	0.76	0.70	0.77	–								
8	Week 10	3.61	0.52	− 0.72	2.89	0.83	0.85	0.67	0.70	0.78	0.73	0.70	0.66	0.75	–							
*Social wellbeing*																						
9	Week 0	3.84	0.99	− 0.37	− 0.41	0.76	0.77	0.34	0.34	0.30	0.22	0.19	0.15	0.25	0.27	–						
10	Week 1	3.94	0.94	− 0.42	− 0.10	0.78	0.79	0.30	0.42	0.34	0.22	0.18	0.16	0.25	0.28	0.75	–					
11	Week 2	3.98	0.89	− 0.38	− 0.05	0.78	0.78	0.19	0.27	0.25	0.16	0.16	0.14	0.18	0.23	0.73	0.74	–				
12	Week 3	3.95	0.89	− 0.27	− 0.42	0.79	0.80	0.30	0.33	0.30	0.22	0.15	0.20	0.24	0.26	0.70	0.76	0.75	–			
13	Week 4	3.98	0.88	− 0.33	− 0.19	0.78	0.80	0.20	0.29	0.23	0.23	0.35	0.19	0.24	0.27	0.63	0.66	0.73	0.77	–		
14	Week 5	4.00	0.90	− 0.26	− 0.41	0.83	0.84	0.25	0.34	0.28	0.21	0.23	0.18	0.26	0.32	0.74	0.74	0.77	0.83	0.78	–	
15	Week 6	3.97	0.95	− 0.34	− 0.47	0.85	0.85	0.26	0.32	0.30	0.22	0.23	0.22	0.28	0.34	0.71	0.70	0.76	0.83	0.78	0.85	–
16	Week 10	4.00	0.93	− 0.36	− 0.15	0.82	0.83	0.25	0.32	0.28	0.24	0.30	0.14	0.22	0.32	0.64	0.68	0.72	0.75	0.75	0.80	0.82

*μ* mean, *σ* standard deviation, *SK* skewness, *Rku* kurtosis, *α* Chronbach’s Alpha, *ρ* Composite Reliability

All values of correlations are statistically significant at *p* < 0.01

### Unconditional LGM

Before estimating and comparing different latent growth models, a series of confirmatory factor analytical models for SSR and SWB, at each measurement instance, were estimated. Here, each item was estimated to load onto its a priori theoretical factorial model. Observed items were used as indicators for latent factors and no items were omitted. With the exclusion of Week 2 and 4 on the SWB scale, no error variances on items were permitted to correlate. Items 2 and 4 for both Week 2 and 4 on the SWB scale were allowed to co-vary to improve overall model fit. The results, summarized in Table 7, showed that the factorial validity for both SSR and SWB at each measurement instance could be established. All factorial models showed acceptable levels of model fit (CFI/TLI > 0.90; RMSEA < 0.08, *p* > 0.05; SRMR < 0.08) and we could therefore proceed to estimate and compare the competing LGMs.

A series of LGM were estimated to find the best fitting model for both SSR and SWB. Comparing an intercept-only, linear-, quadratic-, and piecewise unconditional LGM presented in Table 4 showed that the piecewise latent growth model fitted the data best for both SSR (χ^2^_(27, *N*=175)_ = 37.98, *p* = 0.08, TLI/CFI = 0.99, RMSEA = 0.08) and SWB (χ^2^_(27, *N*=175)_ = 24.97, *p* = 0.58, TLI/CFI = 1.00, RMSEA = 0.00). Two growth trajectories were confirmed for both factors: Phase 1: before the lockdown procedures (Time 0–4) and Phase 2: during lockdown (Time 5–7). The knot was set at Time 4.

**Table 4:** Unconditional Latent Growth Model fit statistics, unstandardized means and -variances

Model	χ^2^	*df*	*p*-value	TLI	CFI	RMSEA	SRMR	AIC	BIC	90% C.I RMSEA		Model Comparison	Δχ^2^	Δ*df*	ΔCFI
										LL	UL				
*Social study resources*															
M0. Intercept Only	64.29	27	0.00	0.94	0.94	0.09	0.09	814.43	868.14	0.061	0.117	M3 vs M0	–26.31	0	0.05
M1. Linear	69.89	31	0.00	0.95	0.94	0.09	0.10	806.28	847.35	0.059	0.112	M2 vs M1	–31.76	–4	0.04
M2. Quadratic	38.13	27	0.08	0.98	0.98	0.05	0.09	779.34	833.04	0.000	0.082	M3 vs M2	–0.15	0	0.01
M3. Piecewise	**37.98**	**27**	**0.08**	**0.99**	**0.99**	**0.08**	**0.08**	**780.44**	**834.14**	**0.000**	**0.080**	M3 vs M1	–31.91	–4	0.04
*Social wellbeing*															
M4. Intercept Only	45.16	27	0.02	0.98	0.98	0.06	0.08	2183.47	2237.17	0.027	0.093	M7 vs M4	–20.19	0	0.02
M5. Linear	35.59	31	0.26	1.00	1.00	0.03	0.07	2162.48	2203.55	0.000	0.066	M6 vs M5	–9.27	–4	0.00
M6. Quadratic	26.32	27	0.50	1.00	1.00	0.00	0.07	2159.46	2213.17	0.000	0.057	M7 vs M6	–1.38	0	0.00
M7. Piecewise	**24.97**	**27**	**0.58**	**1.00**	**1.00**	**0.00**	**0.07**	**2157.66**	**2211.37**	**0.000**	**0.054**	M7 vs M5	–10.62	–4	0.00

*χ*^*2*^ Chi-square, *df* degrees of freedom, *TLI* Tucker-Lewis Index, *CFI* Comparative Fit Index, *RMSEA* Root Mean Square Error of Approximation, *SRMR* Standardised Root Mean Square Residual, *AIC* Akaike Information Criterion, *BIC* Bayes Information Criterion, *LL* Lower Level, *UL* Upper Level, *statistically significant (p < 0.05), ΔX^2^ Chi Square Diff test, Bold text reflects the best fitting model

Further, the unstandardized estimates in Table 5 shows that intercept at baseline (i.e. the average starting value) was significant for both SSR (I_ssr_ = 3.579, S.E. = 0.04, *p* < 0.05) and SWB (I_swb_ = 3.87, S.E. = 0.07, *p* < 0.05). This implies that at the start of the semester students experienced average levels of SSR and SWB. Further, both intercepts’ variances were significant, showing inter-individual variability. Individual growth trajectories significantly differed from one another around the estimated mean for both SSR and SWB.

**Table 5:** Unconditional Piecewise Latent Growth Model results: unstandardized estimates, -means, -variances and t-values

Factor	Social Study Resources			Social Wellbeing		
	Estimate (S.E.)	*t* value	*p* value	Estimate (S.E.)	*t* value	*p *value
*Covariances*						
S1 with I	− 0.012 (.00)	− 3.03	0.00	–0.029 (.02)	− 1.74	0.05
S2 with I	0.001 (.00)	0.52	0.60	0.002 (.01)	0.33	0.74
S2 with S1	− 0.001 (.00)	− 1.53	0.13	0.000 (.00)	− 0.21	0.84
*Means*						
I	3.579 (.04)	92.05	0.00	3.870 (.07)	54.74	0.00
S1	− 0.016 (.01)	− 1.94	0.05	0.016 (.01)	1.28	0.20
S2	0.010 (.01)	2.03	0.04	0.001 (.01)	0.16	0.87
*Variances*						
I	0.224 (.04)	6.20	0.00	0.714 (.09)	7.67	0.00
S1	0.005 (.00)	4.41	0.00	0.011 (.00)	2.87	0.00
S2	0.001 (.00)	1.25	0.21	0.002 (.01)	0.89	0.37

*Statistically significant (*p* < 0.05)

*I* Intercept, *S1* Slope before lockdown, *S2* Slope during lockdown

**Fig. 1:**
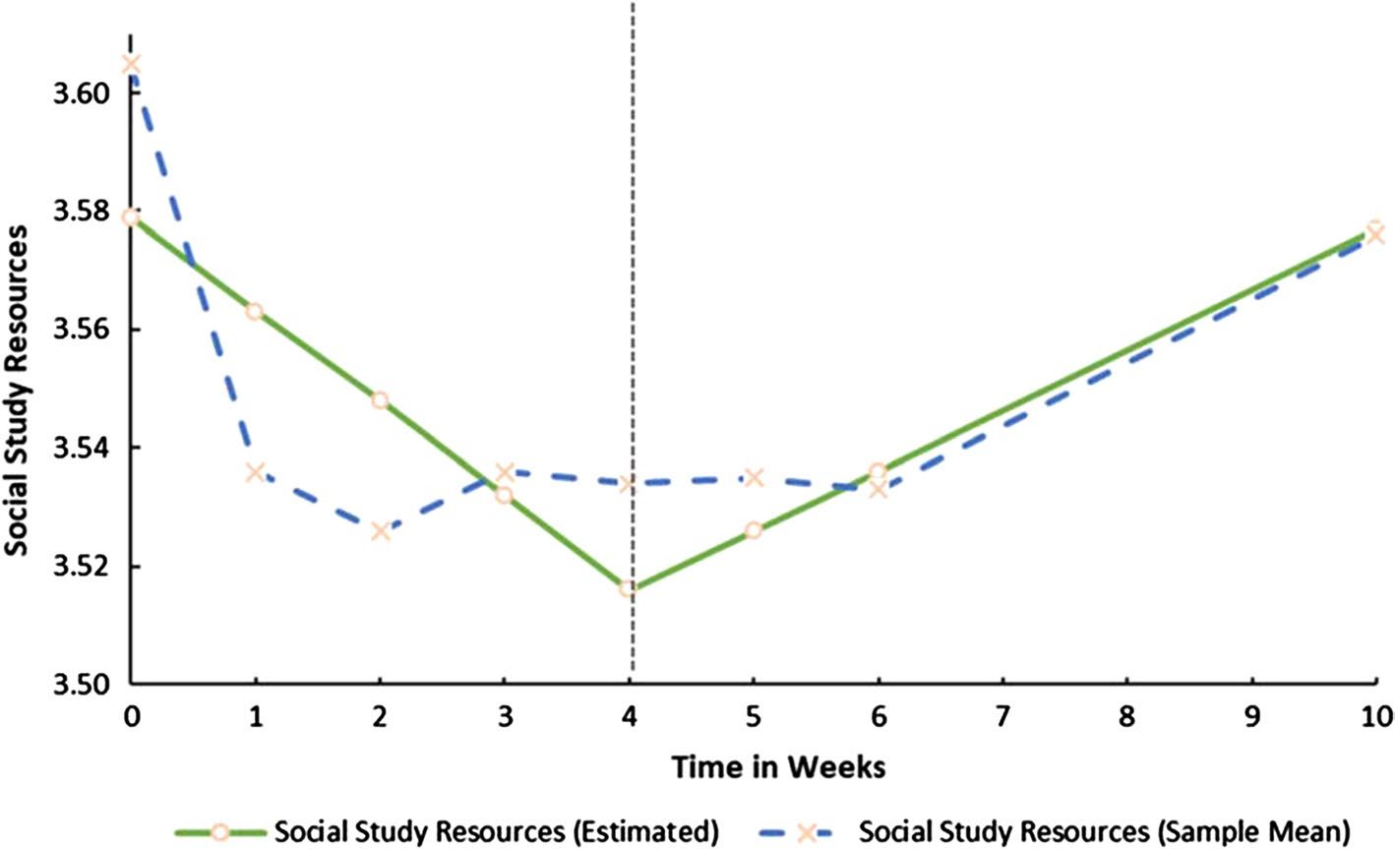
Estimated trajectory of Social Study Resource development before and during COVID-19 Lockdown

**Fig. 2:**
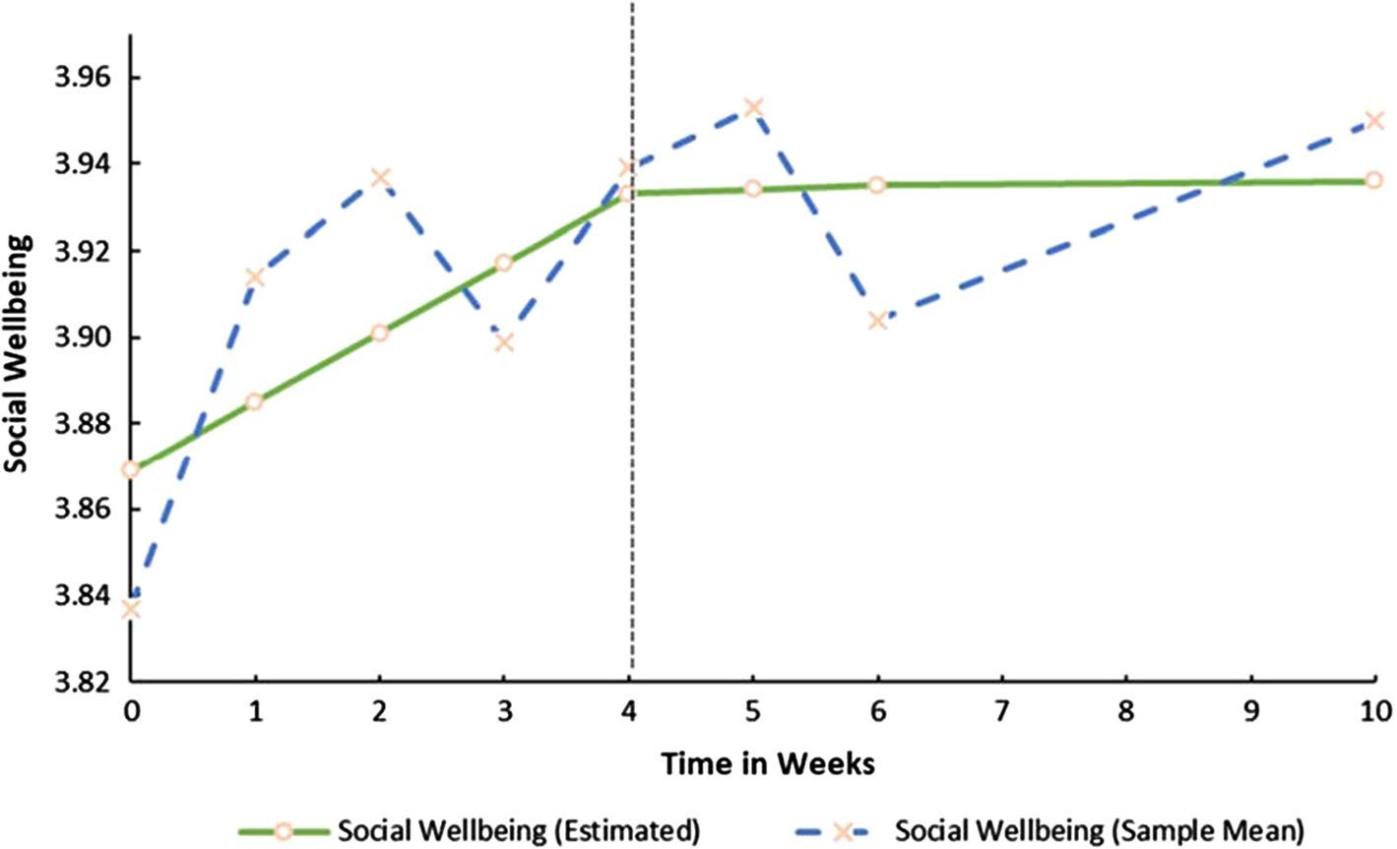
Estimated trajectory of Social Wellbeing development before and during COVID-19 Lockdown

Both growth trajectories (i.e. the Slopes) for SSR were significant. This implies that before the COVID-19 lockdown, SSR decreased linearly (S_1ssr_ = -0.016, *p* < 0.05) but during lockdown increased by 0.01 base points week-on-week (S_2ssr_ = 0.010, *p* < 0.05). However, both the latent growth trajectories for SWB were non-significant (S_1swb_ = 0.016, *p* > 0.05; S_2swb_ = 0.001, *p* > 0.05) implying that it remained relatively constant throughout the study period. Figures 1 and 2 provides a graphical representation of the growth trajectories for both SSR and SWB.

The significant co-variances between the Intercept and Slope 1 of SSR as well as that of SWB indicates that the rate of change in SSR (*Cov*(I_ssr_, S1_ssr_) = -0.012, *p* < 0.05) and SWB (*Cov*(I_swb_, S1_swb_) = -0.029, *p* < 0.05) was significantly negatively related to their respective starting values. In other words, those who reported high at baseline had a slightly faster rate of decline before the lockdown measures.

All the remaining covariances between slopes and intercepts for both the SSR- and SWB models were non-significant (*p* > 0.05) implying that the rate of change during lockdown was not dependent upon their initial values.

### Sequential Piecewise Multi-Process LGM

**Fig. 3:**
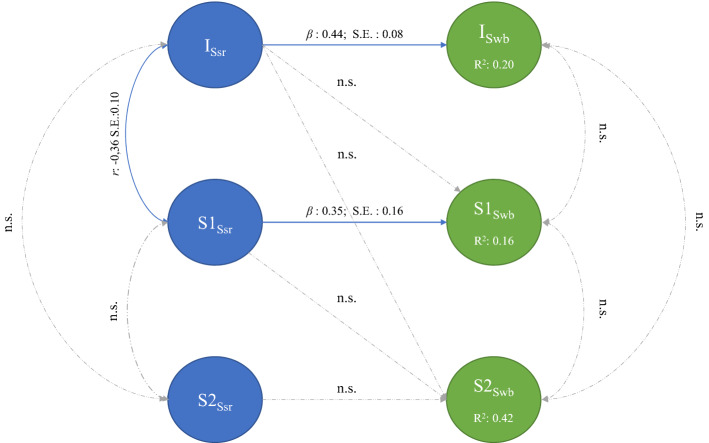
Sequential piecewise multi-process LGM

Figure 3 provides a graphical overview of the sequential piecewise multi-process LGM. This process aimed to sequentially model the growth trajectories and longitudinal associations between SSR and SWB. The results shows that the model produced excellent model fit (χ^2^_(112, *N*=175)_ = 143.91, TLI/CFI = 0.98, RMSEA = 0.04[CI: 0.016-0.059], SRMR = 0.08). The intercept of SSR (I_ssr_) was positively associated with baseline levels of SWB (I_swb_) (*β*: 0.44, S.E.:0.08) which implies initial levels of the former directly influenced initial levels of the latter. Further, the initial growth trajectory of SSR (S_1ssr_) before the COVID-19 lockdown procedures, predicted the initial rate of change in SWB (S_1swb_) (*β*: 0.35, S.E.:0.16).

Finally, only the covariance between the Intercept and pre-lockdown Slope of SSR were significant showing that higher starting levels were associated with a faster decline before lockdown (*Cov*(I_Ssr_, S1_Ssr_) =  −.36, S.E.:0.10). No other associations between the intercepts and slopes for either SSR or SWB was found. The results imply that both SSR and SWB changed at different rates and in different directions after lockdown. Where SSR decreased before- (I_Sr_= 3.579, S1_ssr_ =  −.016 *p* < 0.05) but increased after lockdown (S2_ssr_ = 0.010, *p* < 0.05), SWB stayed moderate and stable before and during lockdown (I_swb_= 3.870, *p* < 0.05, S1_swb_ = 0.016, S2_swb_ = 0.001, *p* > 0.05).

## Discussion

The purpose of this paper was to investigate the trajectory patterns, rate of change and longitudinal associations between SSR and SWB of master students before, and during the intelligent COVID-19 lockdown in the Netherlands. The results were paradoxical and contradicting to initial expectations. Where SSR showed a linear rate of decline before- and significant growth trajectory during the lockdown, SWB remained moderate and stable. Further, initial levels and growth trajectories between SSR and SWB were only associated before the lockdown.

### Growth Trajectories of Social Study Resources and Social Wellbeing

Conventional wisdom assumed that both SSR and SWB would significantly decrease over time due to social isolation during the COVID-19 lockdown; where students’ social needs would not be met and that radical changes in the educational system would result in a larger distance between students and peers as well as between students and lecturers. However, in this study, it was found that neither SSR nor SWB was adversely affected by the lockdown procedures and that the changes in the educational system may have been beneficial.

Despite the COVID-19 lockdown, students followed a relatively traditional developmental trajectory in relation to their experiences of SRR throughout the quartile (Cheng 2020; Landow 2006). Landow (2006) argued that study-related resources, such as peer- and lecturer support, fluctuates throughout a semester, starting high at the beginning and systematically decreasing over-time as work-pressure, deadlines, study-life conflict and stress increases. Given that universities are traditionally understaffed, lecturers’ availability also declines throughout the semester, further limiting access to vital SSR (Jack et al. 2018; Waight and Giordano 2018). However, during vacation periods, students experiences and expectations of these SSR would ‘reset’ and return to normal levels before the next semester (Cohen et al. 2000; Etzion and Zvi 2004). A similar trend was present in our results. SSR growth trajectories declined during the first five weeks of the quartile, coupled with an increase back to normal levels thereafter. During the week of the COVID-19 lockdown announcement, universities in the Netherlands ceased all educational activities (incl. lectures, exams, assignments etc.) for 9 days to transition to a new online educational modus (TU/e, 2020). Despite the uncertainty which may have been associated with this drastic change in tuition methods, students may have perceived these 9 days as a ‘vacation’, allowing for a faster recovery after educational initiatives were re-started.

Further, perceptions of available SSR may also have increased during lockdown for several practical reasons. First, where Dutch universities are traditionally slow in making decisions and communicating such to stakeholders, during the COVID-19 lockdown, universities, as well as lecturers, increased communication frequency on how educational matters would be managed (*c.f.* Maastricht, 2020). Access to lecturers via electronic (e.g. emails, learning management systems) increased significantly, and this may have helped to contain the anxiety associated with the changes in educational activities. Secondly, with the new online educational models being introduced, students may have experienced more autonomy in attending to educational activities (e.g. viewing video lectures when convenient to them), and it may have been easier to negotiate assignment deadlines/exam content with lecturers. This may have strengthened perceptions of lecturer support as educational activities are usually highly structured with limited scope for major changes during the progression of a course (Landow 2006).

The results also showed that the growth trajectory of SWB stayed moderate and stable throughout the quartile. Where studies during the SARS pandemic showed increased levels of loneliness and psycho-social distress during quarantine/social isolation (Brooks et al. 2020; Lau et al. 2005), the same trend was not present in the current sample. Zhang and Ma (2020) reported similar trends in China during the COVID-19 outbreak, showing no changes in mental health and social wellbeing of participants. Fried (2020) replicated these findings showing no major changes in emotional, psychological, or social wellbeing during the COVID-19 lockdown in the Netherlands. Both studies showed that that the negative psychological and/or social impact of the lockdown procedures were largely negated due to individuals spending more time outdoors, and with loved ones (on- and offline), engaging in more pleasurable activities (such as trying out new hobbies), resting/relaxing more and receiving more social support from friends and family members (Fried 2020; Zhang and Ma 2020). Students in the Netherlands also showed no changes in their in-person social activities (Fried 2020). The COVID-19 lockdown may also have made students more aware of their SWB and social needs due to increasing media attention around the matter, therefore resulting in active efforts to manage such healthily and sustainably (Lades et al. 2020). As such, despite the possible fear, apprehension and anxiety which may be resultant from the COVID-19 lockdown, students may be more active in identifying, managing and addressing their social needs (Ebrahim et al. 2020; Zhang & Ma, 2020).

Two final contextual factors may also provide an explanatory narrative as to why SSR and SWB were not negatively affected by the COVID-19 lockdown. *Firstly,* all participants in this study were first-year master *students*. Master students are more autonomous, agile, and resourceful than their undergraduate peers (Cilliers and Flotman 2016; El-Ghoroury et al. 2012). Master students have pre-established (functional) social support networks within the university and know how/when to access the necessary social resources required to perform academically (Cilliers and Flotman 2016). These students are also known to present with higher levels of academic self-efficacy and may have crystalised coping mechanisms to manage both study-related- and environmental demands (Jorgensen et al. 2016; Nor and Smith 2019). Master students may therefore be less susceptible to the impact of the lockdown procedures.

*Secondly*, the package of NPIs introduced by the Dutch Government may also have played a role. Unlike other countries where individuals were confined to their homes, the Intelligent COVID-19 lockdown measures allowed for in-person interaction and socialisation (under the 1.5-m social distancing restriction) (de Haas et al. 2020). Students were therefore free to meet and spend time with their friends and family. Fried (2020) reported that during the first weeks of the COVID-19 lockdown within the Netherlands, students did not report any changes to the frequency or amount of social interactions they had with friends or family. Fried (2020) also reported that the levels of loneliness students experienced decreased dramatically over the first two weeks of the lockdown. Therefore, despite the restrictions being in place, students were still able to meet their social needs which may have negated the potential negative impact of the COVID-19 Lockdown on their SWB.

### Longitudinal Associations Between Growth Trajectories

Finally, the paper aimed to determine the longitudinal association between the growth trajectories of SSR and SWB but found that such only existed before lockdown procedures were implemented. SSR at baseline was positively associated with that of SWB, implying that those how perceived to have greater access to peer- and lecturer support at the beginning of the quartile, may be more likely to experience higher levels of SWB (Cilliers et al. 2018; Lesener et al. 2020). Similarly, the rate of change in SSR also directly affected the growth trajectory of SWB before lockdown. This implies that as SSR increases under normal circumstances, that it affects the rate at which SWB develops over time. However, during the lockdown, the results showed that SSR and SWB developed at their own rates and in separate directions. Taken together, it seems as though the relationship between the factors follows a traditional trajectory and association before lockdown, where higher levels of the former, leads to higher reports in the latter.

Further, during the lockdown, the associations are perceived differently by students. This may be due to the sudden changes in the educational setup and the strategies the university employed to mitigate the impact thereof on students, which in turn directly affected SSR. Students were provided with real-time information with regards to decisions impacting education, lecturers were more supportive and understanding in relation to the challenges students faced and were more accommodative in respect of assignment deadlines. However, these strategies were not specifically targeted at enhancing the SWB of students. Changes at university were implemented with the intent to continue the educational programme, with specific focus being placed on easing the transition from offline to online education (Maastricht, 2020; TU/e 2020). Universities were therefore less focused on the mental health and SWB of students. Given these reported increases in SSR and the relative stability of SWB during the lockdown, the association between the two factors disappeared.

### Study Limitations

The study had several limitations which affect the interpretation of the results. First, the COVID-19 pandemic and the associated lockdown occurred during the data collection process of an international student wellbeing project. This implies that the study could not control for specific COVID-19 related factors such as fear of infection.. Therefore, the specific COVID-19 related moderators or attributing factors which could have impacted SSR and SWB during the lockdown was not measured. Second, the sample was drawn from a single cohort of master students at a specific university in the Netherlands (which was at the heart of the initial COVID-19 outbreak in the Country). Therefore, the experiences reported may have differed from those of other universities in the country. These results may therefore not be generalizable. Thirdly, the measurement instances during lockdown only spanned a few weeks, and students may still have been coming to terms with the “new normal”. Therefore, additional measures during lockdown may have painted a more holistic picture. Fourthly, the study focused on group related changed and did not explore specific inter-individual differences.

**Table 6:** Estimated statistical power by sample size using the Satorra-Saris method for testing rate of change in LGM

Sample Size	λ	Power
50	4.552	0.56
60	5.445	0.64
70	6.338	0.71
80	7.231	0.76
90	8.123	0.81
100	9.016	0.85
110	9.909	0.88
120	10.802	0.90
130	11.695	0.92
140	12.588	0.94
150	13.481	0.95
160	14.374	0.96
170	15.267	0.97
175	15.714	0.97

λ Estimated noncentrality parameter

**Table 7:** Model fit statistics for individual confirmatory factor analytical models for Social Study Resources and Social Wellbeing

Model	χ^2^	*df*	*p*-value	CFI	TLI	RMSEA	SRMR	AIC	BIC	90% C.I RMSEA	
										LL	UL
*Social study resources*											
Week 0	53.65	26	0.00	0.93	0.90	0.08	0.06	3466.80	3554.77	0.048	0.109
Week 1	51.21	26	0.00	0.93	0.90	0.08	0.07	3155.99	3243.30	0.045	0.107
Week 2	54.87	26	0.00	0.93	0.91	0.08	0.06	2955.09	3041.20	0.052	0.114
Week 3	64.49	26	0.00	0.90	0.90	0.08	0.06	2836.88	2922.45	0.068	0.127
Week 4	32.64	26	0.17	0.98	0.98	0.04	0.05	2839.04	2923.71	0.000	0.080
Week 5	37.14	26	0.07	0.98	0.97	0.05	0.05	2788.57	2873.79	0.000	0.088
Week 6	41.92	26	0.03	0.96	0.95	0.06	0.06	2689.68	2774.53	0.023	0.097
Week 10	56.22	26	0.00	0.93	0.90	0.08	0.06	2681.18	2765.48	0.056	0.120
*Social wellbeing*											
Week 0	12.56	5	0.03	0.96	0.91	0.08	0.04	2789.81	2836.93	0.029	0.161
Week 1	9.68	5	0.08	0.97	0.93	0.08	0.04	2588.23	2635.00	0.000	0.145
Week 2	2.48	3	0.48	1.00	1.00	0.01	0.02	2372.18	2424.46	0.000	0.124
Week 3	12.60	5	0.03	0.96	0.91	0.08	0.05	2297.54	2343.39	0.030	0.168
Week 4	2.15	3	0.34	0.99	0.99	0.02	0.02	2231.26	2285.69	0.000	0.164
Week 5	10.01	5	0.04	0.97	0.91	0.08	0.04	2161.93	2210.62	0.019	0.177
Week 6	3.15	5	0.68	1.00	1.00	0.01	0.02	2134.20	2179.67	0.001	0.088
Week 10	7.33	5	0.20	0.99	0.98	0.06	0.03	2161.63	2206.79	0.000	0.136

*χ*^*2*^ Chi-square, *df* degrees of freedom, *TLI* Tucker-Lewis Index, *CFI* Comparative Fit Index, *RMSEA* Root Mean Square Error of Approximation, *SRMR* Standardised Root Mean Square Residual, *AIC* Akaike Information Criterion, *BIC* Bayes Information Criterion, *LL* Lower Level, *UL* Upper Level

## Conclusion

This paper is the first to look at how trajectories of social wellbeing and social student resources change before and during the COVID-19 outbreak in the Netherlands. It provides valuable information which could be used to understand how pandemics actively affect components of mental health. The findings present an alternative view of the current narrative within the literature as to the negative impact of the COVID-19 pandemic on the social wellbeing of students.

## Appendix

See Tables 6 and 7
